# Exploring knowledge, perception, and use of surface electromyography in physiotherapy post graduate trainees in Italy: a single center preliminary survey

**DOI:** 10.3389/fresc.2024.1489927

**Published:** 2024-10-22

**Authors:** Gianluca Bertoni, Gaia Leuzzi, Mirko Job, Marica De Simone, Marco Testa

**Affiliations:** ^1^ Department of Neurosciences, Rehabilitation, Ophthalmology, Genetics, Maternal and Child Health (DINOGMI), University of Genova, Genova, Italy; ^2^ Department of Clinical and Experimental Sciences, University of Brescia, Brescia, Italy; ^3^ Training Unit, Azienda Socio-Sociosanitaria Territoriale di Cremona, Cremona, Italy; ^4^ Department of Physical Education and Rehabilitation, Experimental Anatomy Research Group (EXAN), Vrije Universiteit of Brussel (VUB), Brussels, Belgium

**Keywords:** survey, physiotherapy, surface electromyography (sEMG), Rehabilitation, electromyography

## Abstract

**Introduction:**

Surface electromyography (sEMG) is a non-invasive technique that records muscle electrical activity using skin-surface electrodes, aiding physiotherapists in assessing and treating muscular and neuromuscular conditions. Despite its potential, sEMG remains underutilized in Italy. This study aims to evaluate Italian physiotherapists’ knowledge and use of sEMG, specifically among those who completed the Master's Degree in Rehabilitation of Musculoskeletal and Rheumatological Disorders at the University of Genoa.

**Methods:**

This cross-sectional study, approved by the University of Genoa's Ethical Committee, utilized an anonymous web survey to gather data from physiotherapy students in the master's program. The survey, developed based on the International Handbook of Survey Methodology, consisted of 12 questions covering demographics, previous sEMG experience, the importance of sEMG in practice and research, and educational satisfaction. Data collection spanned from January to May 2024, with a response rate of 72.7% (93 participants). Descriptive analysis was used to summarize the data.

**Results:**

The average age of respondents was 26.5 years, with 55.9% being male. Only 3.2% reported using sEMG in their practice. While 46.2% considered sEMG moderately important for practice, 40.9% deemed it extremely important for research. Most participants felt their undergraduate education inadequately prepared them for using sEMG, with 81.7% rating their preparation as insufficient. Although the master's program improved sEMG knowledge, 66.7% indicated no significant proficiency gain.

**Conclusion:**

Italian physiotherapists view sEMG mainly as a research tool rather than a clinical one. The findings highlight the need for curriculum reforms to enhance both theoretical and practical sEMG education. Simplifying and standardizing sEMG protocols and integrating sEMG training into physiotherapy curricula are essential steps to better prepare clinicians for its clinical application.

## Introduction

Surface electromyography (sEMG) is a non-invasive diagnostic technique that records the electrical activity of muscles through electrodes placed on the skin's surface (1). This tool provides physiotherapists with a valuable window into muscle function, allowing them to assess and improve the treatment of a wide range of muscular and neuromuscular conditions (2).

sEMG detects the electrical currents generated by motor units during muscle activity. Motor units consist of a motor neuron and all the muscle fibers it innervates (3). In order to contract a muscle, the nervous system sends electrical signals to the motor units, causing depolarization of the muscle fibers and generating an action potential (3). These signals can be detected by surface electrodes, translated into electrical signals, and recorded to be analysed (3).

Unlike needle EMG which requires the insertion of needle electrodes into the muscle by a specialized professional, sEMG is a non-invasive procedure well tolerated by patients and it can be performed by any health professional. Electrode placement can be quickly adapted to different muscle areas, allowing for comprehensive and detailed evaluation.

This tool is particularly useful in various clinical applications, as it allows to evaluate the physiological changes in the muscle activity during voluntary and elicited contraction (i.e., amplitude and myoelectric manifestations of fatigue) (3, 4) and to document change in neuromuscular function during or after therapy interventions (5, 6). sEMG can be used to evaluate the timing of muscle activity, understanding their coordination and responses in particular musculoskeletal conditions. For instance, alterations in the activation pattern of the Erector Spinae muscle have been consistently reported in people with chronic low back pain and have been associated with self-reported disability (7–9), pain (10–12), and fear of pain and re-injury (12).

Physiotherapists can use sEMG to evaluate muscle function and design personalized rehabilitation programs (2), monitoring and guiding the effectiveness of exercises, ensuring that patients correctly perform prescribed exercises and providing real-time feedback (3, 8, 13–15). Therefore, sEMG proves to be a potentially valuable tool for physiotherapists, offering detailed analysis of muscle activity and enabling a more targeted and effective therapeutic approach to various challenges in muscle rehabilitation (16).

However, sEMG is currently not well known and scarcely used by physiotherapists in Italy, both in research and clinical practice (2, 17). This limited dissemination can be attributed to several factors, including a lack of awareness regarding the potential of this tool in the evaluation and treatment of neuromuscular issues (2, 17). Recognizing the need to bridge this gap and promote greater adoption of sEMG, we propose a study focused on analyzing the opinions of a specific population of Italian physiotherapists. This study will be conducted among those who participated in the training program of the 20th edition of the Master's Degree in Rehabilitation of Musculoskeletal and Rheumatological Disorders at the University of Genoa (18). The aim is to assess the perceptions and knowledge acquired by these professionals during the training course, exploring the real possibilities of integrating sEMG into their clinical practice and in possible rehabilitation research contexts. Gathering this information aims to encourage wider dissemination of this innovative technology among Italian physiotherapists, promoting a more advanced and personalized approach to managing muscular and neuromuscular conditions.

The objective of this project is to investigate the knowledge and use of surface electromyography in the clinical practice of physiotherapists.

## Methods

Ethical approval was granted by the University's Ethical Committee for Research of the University of Genoa (CERA2024.18, approved February 22, 2024). The conduction of this study respected the Declaration of Helsinki (19). We followed the Strengthening the Reporting of Observational Studies in Epidemiology (STROBE) recommendations for the reporting of this study (20).

### Survey tool development and setting

This cross-sectional study adopted an anonymous web survey instrument developed according to the “International Handbook of Survey Methodology” (21). A panel of different professionals, including a physiotherapist (GB), a bioengineering (MJ) and a sports scientist (GL), following a brainstorming, created the first survey instrument. The draft was presented to MT and a pool of participants to test its relevance and understandability. Based on their feedback, the survey instrument was adjusted to reach the final draft and disseminate it online using “Microsoft Forms Suite Office 365”, which is a safe application that respects the European General Data Protection Regulation (22). To ensure complete anonymity, the survey link was distributed to students by the administrative office of the master's program, external to the research team. The system did not collect respondents’ email addresses, meaning that the authors were never privy to any identifying information about the participants, thereby fully safeguarding their anonymity.

### Web-survey instrument

The survey instrument investigated the use of, and education on sEMG in clinical practice among physiotherapists. The instrument, with a total of 12 questions, was structured into four sections. The first section, including the first four questions, covered the sociodemographic characteristics of the sample (i.e., age, gender, years of clinical experience, and workplace). The second section included only a question (number five) that investigated if participants had any previous experience with sEMG in clinical practice. The third section, from questions six to eight, investigated the importance of sEMG instrument and competencies in clinical practice and research. The fourth and final section, from questions nine to 12, investigated the educational level and satisfaction with sEMG instrument. In the third and fourth sections, the participants were asked to answer by using a 4-point Likert scale ranging from 1 (lowest score) to 4 (highest score), with explanations provided for each possible answer. The neutral option was removed to encourage participants to take a clear stance and have deeper engagement with the questions (23). The full survey is available in the Supplementary File 1.

### Participants

This online survey instrument was addressed to Italian physiotherapy students of the master's degree program in musculoskeletal (MSK) disorders rehabilitation at the University of Genova, Italy (Master RDM) (18). The participants in this master's program are already qualified physiotherapists, holding a 3-year Bachelor of Science degree and regularly practicing in Italy, who have chosen to further their education with a post-graduate specialization (18).

This master's program included an introduction to electromyography, although the course content was purely theoretical. The general training on sEMG consisted of 8 h, covering topics such as basic electrode placement, signal acquisition, interpretation, and potential clinical applications of sEMG (24). Participants, prior to participate in the study, were asked both to read an informative note about the study and the data treatment and to provide their informed consent. To complete the survey, 4 min were required. Students were reached through various channels, including newsletters, social media advertisements, and direct face-to-face invitations. Filling the survey was voluntary and no incentives were provided. No exclusion criteria were applied, as the study aimed to focus on a specific cohort of physiotherapists who had undergone the same theoretical training on sEMG.

### Data analysis

For the data analysis, continuous variables were presented as mean values ± standard deviations (SD), while categorical variables were presented as absolute values and frequency percentages. Descriptive analysis was adopted for socio-demographic data (section one), to give an overall picture of our sample. Data from section two were reported as raw numbers of absolute values and frequency percentages. Responses to sections three and four were graphically presented reporting the percentage and number of each Likert item. Participants who partially or totally agreed on a Likert scale (score 3–4) were considered to agree with the statements. Microsoft Forms does not allow recording participants’ data unless they answered all the questions, therefore, there were no missing data.

## Results

We collected data from February to May 2024, for a total of 93 surveys that were further included in the analysis (response rate of 72.7%). The participants’ mean age was 26.5 ± 2.8 years, with the majority being men (52 men, 55.9%) and 41 women. Table 1 reports the description of the sample.

**Table 1:** Demographics characteristics and previous use of sEMG.

	Mean (SD)
*N* = 93	
Age	26.5 (2.8)
Years of working experience as a physiotherapist	3.2 (2.5)
Gender	*N* (%)
Male	52 (55.9%)
Female	41 (44.1%)
Workplace	*N* (%)
Hospital	12 (9.7%)
Private practice	63 (50.8%)
Rehabilitation clinic	36 (29.1%)
Unoccupied	1 (0.8%)
Professional sports team	1 (0.8%)
Home care	6 (4.8%)
Nursing home	5 (4.0%)
Use of sEMG	
Yes	3 (3.2%)
No	90 (96.8%)

N, total number; SD, standard deviation.

**Figure 1:**
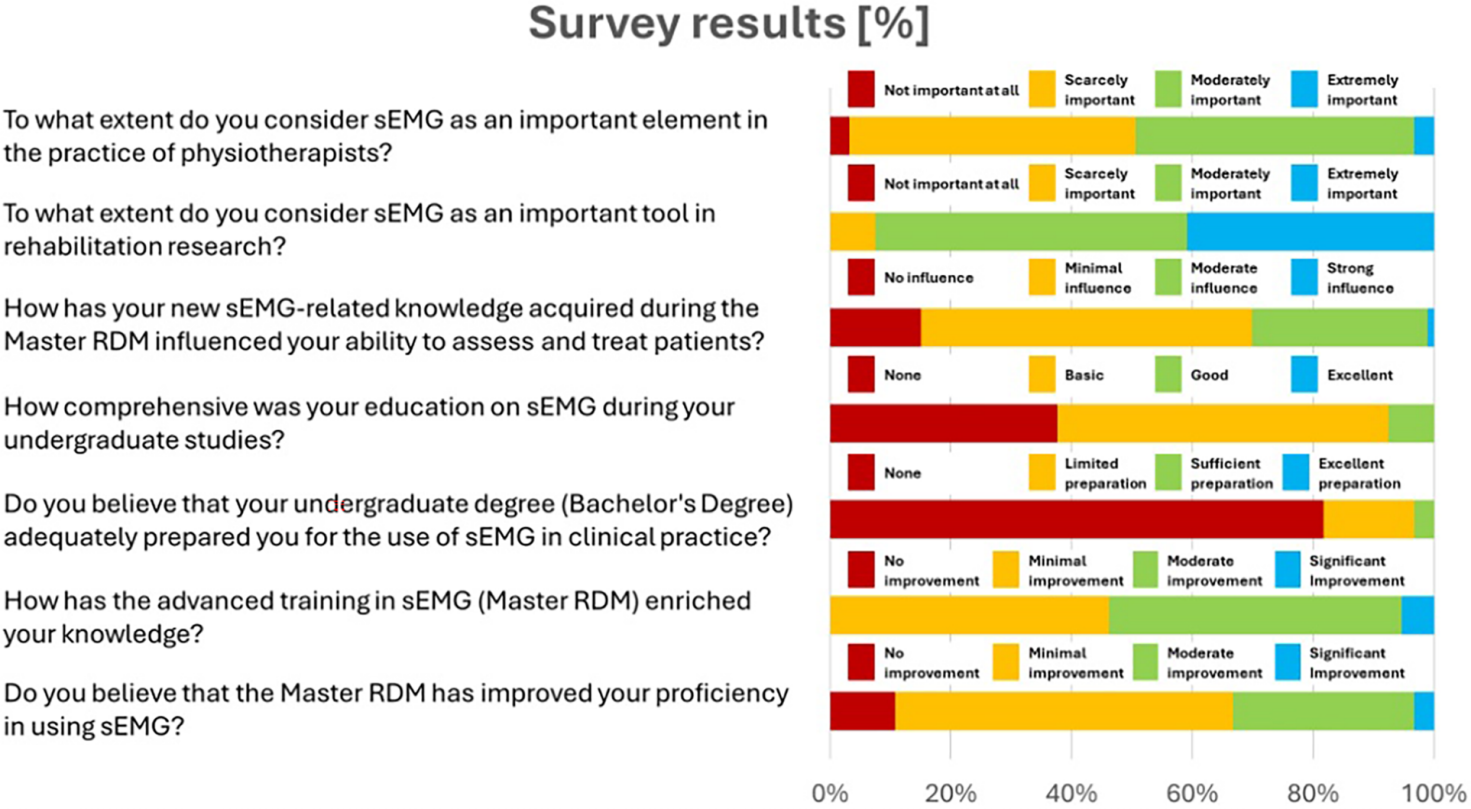
Responses to the 7 key questions on the importance of sEMG and its educational impact on physiotherapists.

Hereafter, Table 2 shows the percentage of responses for each answer for each of the remaining questions. Figure 1 graphically represents the same results for a clearer overview of the distribution of responses for each item.

**Table 2:** Distribution of responses for sEMG questions.

Survey item	Survey answers [%] (*N* = 93)			
To what extent do you consider sEMG as an important element in the practice of physiotherapists?	Not important at all	Scarcely important	Moderately important	Extremely important
	3.2%	47.3%	46.2%	3.2%
To what extent do you consider sEMG as an important tool in rehabilitation research?	Not important at all	Scarcely important	Moderately important	Extremely important
	0.0%	7.5%	51.6%	40.9%
How has your new sEMG-related knowledge acquired during the Master RDM influenced your ability to assess and treat patients?	No influence	Minimal influence	Moderate influence	Strong influence
	15.1%	54.8%	29.0%	1.1%
How comprehensive was your education on sEMG during your undergraduate studies?	None	Basic	Good	Excellent
	37.6%	54.8%	7.5%	0.0%
Do you believe that your undergraduate degree (Bachelor's Degree) adequately prepared you for the use of sEMG in clinical practice?	Insufficient preparation	Limited preparation	Sufficient preparation	Excellent preparation
	81.7%	15.1%	3.2%	0.0%
How has the advanced training in sEMG (Master RDM) enriched your knowledge?	No improvement	Minimal improvement	Moderate improvement	Significant improvement
	0.0%	46.2%	48.4%	5.4%
Do you believe that the Master RDM has improved your proficiency in using sEMG?	No improvement	Minimal improvement	Moderate improvement	Significant improvement
	10.8%	55.9%	30.1%	3.2%

## Discussion

This study explored the knowledge and use of the sEMG technique among Italian physiotherapists who completed the master's degree program in Rehabilitation of Musculoskeletal and Rheumatological Disorders at the University of Genova (18).

Our results indicate that the physiotherapists involved in the study do not use sEMG in their clinical practice, viewing it primarily as a research tool. Starting from this perspective, the specific responses revealed several key insights and raised important questions about the application of sEMG in rehabilitation, as well as the potential barriers to its integration into standard clinical practice.

Despite having received general training on sEMG during their master's degree curriculum, more than half (66.7%) of the respondents felt that their proficiency in this technique did not significantly improve after completing the course. The specific module was mostly theoretical, focusing on the basics of electromyography and involving the reading and discussion of research articles. The training primarily focused on physiology, with minimal emphasis on signal processing and interpretation. Thus, the lack of technical and practical content might have contributed to the respondents’ perception of having low competence in sEMG. Our findings align with previous research by Manca et al. (25) who identified slow dissemination of research findings and a lack of education as primary barriers to the clinical use of sEMG in neurorehabilitation. This suggests a broader issue of insufficient training and knowledge transfer, which also emerged in our survey, particularly regarding physiotherapists’ perception of sEMG as a research tool rather than a clinical resource (25). While theoretical knowledge is essential, hands-on experience is equally important for mastering a complex tool like sEMG, and there is a recognised need for increased technical training for both clinical operators and their educators (17). Nonetheless, current physiotherapy academic programs have proven inadequate in equipping clinicians with sufficient expertise in sEMG and other advanced measurement systems, highlighting the urgent need for change (26, 27).

Despite the efforts of professional societies such as the International Society of Electrophysiology and Kinesiology (ISEK) and journals like the Journal of Electromyography and Kinesiology (JEK), which have published tutorials and consensus papers on the use of sEMG, these initiatives have not yet yielded significant improvements in clinical practice. It is unclear whether physiotherapists and their educators are fully aware of these resources or if the current educational frameworks allow for their effective integration. This highlights the need for more proactive engagement by educational institutions and professional organizations to demand and provide better education in this area by encouraging the broader incorporation of these materials at both the undergraduate and postgraduate levels, which could help address existing gaps and foster a more technically proficient workforce.

In this sense, the work of Snöljung and colleagues highlighted the responsibility of undergraduate education in teaching the use of different measuring instruments with an open-minded approach toward progress and advances in evidence-based practice (27). The results obtained from our sample pointed out a very different situation, where undergraduate studies seem to have failed to provide physiotherapists with the necessary knowledge and skills to apply electromyography techniques in clinical practice. Specifically, 96.8% of the participants reported that their undergraduate formation provided insufficient to limited preparation for using sEMG in clinical settings, and 37.6% did not even encounter this topic during their bachelor's degree program. Physiotherapy and kinesiology educational programs are diversely structured worldwide and may include sEMG in their curricula. However, this topic is often not covered in sufficient detail to promote a confident and independent use of sEMG in clinical practice (28). Combined with the use of technical language and concepts typical of the engineering field, this may contribute to sEMG being perceived by physiotherapists as a specialized subject with limited clinical applicability from the early years of their formation. This hypothesis is supported by our findings, which show that a significant percentage of our respondents share the opinion that sEMG is considered moderately (51.6%) to extremely (40.9%) important for research rather than for clinical practice. This result is further validated by the feedback from 69.9% of the participants, who, after attending the master's program's specific courses, believe that the knowledge gained on sEMG has little to no influence on patients’ assessment and treatment.

Overall, the snapshot captured by our survey aligns with the global perspective highlighted in the literature, where despite a large body of research on the subject, the clinical acceptability of sEMG among physiotherapists remains low (29). The primary cause behind this issue is multifaceted, involving cultural, educational, technical, and administrative barriers that limit the widespread clinical use of sEMG in physiotherapy (17).

As highlighted by Manca et al. (25), despite the barriers to adoption, sEMG holds significant clinical utility in patient assessment and treatment optimization. This reinforces the need for better educational initiatives and streamlined protocols to integrate sEMG into routine clinical practice, which was also emphasized by our respondents (25). Advanced modern software can efficiently identify clinically significant features from myoelectric signals and aid their interpretation. Nonetheless, such tools cannot correct human errors such as electrode misplacement or improper experimental settings. Therefore, establishing standardised protocols is a key step toward the successful implementation of sEMG into clinical practice. In this regard, important steps were made to deliver tutorials and guidelines to clinical operators (1, 30–34). For instance, some notable initiatives are the guidelines and tutorials offered through projects like “Surface Electromyography for Non-Invasive Assessment of Muscles (SENIAM)” (32) and “Consensus for Experimental Design in Electromyography (CEDE)” (35). Despite these efforts, their impact has been limited, indicating that while necessary, standardised protocols alone may not be sufficient for widespread adoption of sEMG in clinical practice (36).

Over the past 4 years, despite the work of Manca et al. (25) and advancements in technology, the integration of sEMG into clinical practice by physiotherapists remains limited. The lack of substantial change may be attributed to both the conservative nature of physiotherapy education and the slow pace at which new technologies are adopted. Looking forward, it is expected that future educational reforms, spearheaded by academic institutions and professional bodies, will encourage more extensive use of sEMG in clinical settings. However, such changes will require collaboration across disciplines.

Recent discussions in healthcare, particularly in fields that integrate medical and engineering expertise, suggest that the skills required to operate and interpret advanced technologies such as sEMG may be better suited to professionals trained specifically in clinical technology or rehabilitation engineering. In countries like the Netherlands, the role of clinical technologists has gained recognition, as they bridge the gap between medical and technical domains, allowing for more effective use of cutting-edge tools in clinical practice. This trend raises the question of whether the introduction of similar roles in the rehabilitation field could alleviate the educational burden on physiotherapists, whose curriculum is already densely packed with clinical competencies. Furthermore, these specialized figures could take on the responsibility of managing complex measurements and technological applications, allowing physiotherapists to focus more on clinical decision-making and patient care.

Rehabilitation engineers or clinical technologists, already present in other fields, may become essential in managing technology and performing complex measurements in rehabilitation clinics, thereby relieving physiotherapists of these technical demands.

A key limitation of this research is the sampling of participants from a single institution, specifically the Master's Degree Program in Rehabilitation of Musculoskeletal and Rheumatological Disorders at the University of Genoa. This choice was driven by the aim of preliminarily measuring the perception of a population of physiotherapists who had been introduced to the sEMG methodology. In this sense, as a group of authors, being familiar with the structure of the master's program and its educational and training offerings, we decided to initially limit our analyses to this specific sample. However, this narrow scope inherently limits the generalizability of our findings. Participants’ views may not fully reflect those of physiotherapists trained in different institutions, particularly those who have not received formal instruction in sEMG. The absence of responses from professionals with diverse educational backgrounds prevents us from drawing broader conclusions about the overall adoption and use of sEMG among Italian physiotherapists.

In future studies, we aim to expand the scope by including a more extensive and diverse sample of physiotherapists from different educational and clinical backgrounds.

Another significant limitation arises from the survey's design, which did not include questions regarding the technology's user-friendliness, costs, or its integration into the clinical workflow. As this study served as a preliminary step to assess the relevance of sEMG training in the physiotherapists’ educational framework, these aspects were not considered during the questionnaire's development, since they typically become more prominent in practical and administrative contexts. The cost of sEMG equipment, ranging from approximately $10,000 to $40,000, could be prohibitive for rehabilitation professionals, particularly those in private practice, outside the financial support of larger research and healthcare centers. Additionally, the standard length of a physiotherapy session, typically 30–60 min, limits the opportunity to incorporate the time-intensive sEMG technique into traditional clinical procedures (29, 37, 38). These challenges, combined with physiotherapists’ unfamiliarity with sEMG hardware and related signal-processing techniques (5), are recognized as significant barriers to its implementation in clinical practice. Therefore, future iterations of this research should include questions addressing user-friendliness, workflow integration, and the diagnostic or therapeutic relevance of sEMG. Expanding the scope in this way will help better assess the generalizability of the findings and capture additional factors—such as cultural, administrative, and technical barriers—that may impact the use of sEMG in clinical settings. Furthermore, the instrument's reliability and validity will be formally tested in future iterations to ensure it meets the necessary standards for broader clinical application.

Despite these limitations, physiotherapists play a crucial role in developing and promoting new technological applications in clinical settings for the evaluation, monitoring, and treatment of various movement disorders. In light of this consideration, relying on the findings of our survey, the successful transfer of sEMG techniques into clinical practice necessitates the simplification and standardisation of protocols and signal analysis methodologies through the integration of both engineering and clinical expertise and terminologies. Furthermore, the use of sEMG should be introduced as an integral part of the academic training curriculum of physiotherapy, to strengthen clinicians’ practical knowledge of this technique and ensure that it is reflected in clinical procedures.

## Conclusion

Our findings highlighted that Italian physiotherapists do not routinely use sEMG in clinical practice, as they generally perceive it more as a research tool rather than as a resource for everyday clinical applications. There is a clear necessity for curriculum reforms that enhance both theoretical and practical sEMG education, along with the simplification and standardization of protocols. These changes are crucial to adequately prepare clinicians to utilize sEMG effectively in their practice.

## Data Availability

The raw data supporting the conclusions of this article will be made available by the authors, without undue reservation.
