# Supplementary material for: Exploring knowledge, perception, and use of surface electromyography in physiotherapy post graduate trainees in Italy: a single center preliminary survey
Source: Front Rehabil Sci. 2024 Oct 22;5:1489927. doi: 10.3389/fresc.2024.1489927 (PMC11534621; doi:10.3389/fresc.2024.1489927)
Supplement: Supplementary file 1 [file Table1.docx]

**Survey on the Use of Electromyography in Clinical Practice of Physiotherapists**

Dear colleague of the XXth edition of the RDM Master's program,
Thank you for participating in this survey aimed at evaluating the importance and usefulness of surface electromyography (sEMG) in the clinical practice of professionals and rehabilitation research. Your contribution is of fundamental importance to understand how EMG influences daily clinical practice and rehabilitation research to assess the effectiveness of the training received during your studies, both in the undergraduate degree and in the specialized master’s program. Please remember that all your answers are confidential and will be treated anonymously. Without your valuable contribution, it would not be possible to obtain a complete picture of the impact of EMG in the physiotherapy community. Thank you for your time and cooperation.

Best regards,
Prof. Marco Testa & REHElab Research Team – Rehabilitation Engineering Laboratory

**1^st^ Section: Demographics**

1. Age:
2. Gender:
3. Years of working experience as a physiotherapist:
4. Workplace:

**2^nd^ Section: Previous Experiences:**

1. Have you ever used Surface Electromyography (sEMG) in your clinical practice?
    Yes No

**3^rd^ Section: Importance of sEMG**

1. To what extent do you consider sEMG as an important element in the clinical practice of physiotherapists?

Not important at all Scarcely important Fairly important Extremely important

1. To what extent do you consider sEMG as an important tool in rehabilitation research?

Not important at all Scarcely important Fairly important Extremely important

1. How has your new sEMG-related knowledge during the Master RDM influenced your ability to assess and treat patients?

No influence Minimal influence Moderate influence Strong influence

**4^th^ Section: Education on sEMG**

1. How comprehensive was your education on sEMG during your undergraduate studies?

None Basic Good Excellent

1. Do you believe that your undergraduate degree (Bachelor’s Degree) adequately prepared you for the use of sEMG in clinical practice?

Insufficient preparation Limited preparation Sufficient preparation Excellent preparation

1. How has the advantage training in sEMG (Master RDM) enriched your knowledge?

No improvement Minimal improvement Moderate improvement Significant improvement

1. Do you believe that the Master RDM has improved your proficiency in using sEMG?

No improvement Minimal improvement Moderate improvement Significant improvement
